# Cholesin receptor signalling is active in cardiovascular system-associated adipose tissue and correlates with SGLT2i treatment in patients with diabetes

**DOI:** 10.1186/s12933-024-02322-y

**Published:** 2024-06-20

**Authors:** Aleksandra Ryk, Anna Marcinkiewicz, Jędrzej Chrzanowski, Arkadiusz Mariusz Michalak, Izabela Dróżdz, Jacek Burzyński, Michał Krejca, Wojciech Fendler

**Affiliations:** 1 Department of Biostatistics and Translational Medicine, Medical University of Lodz, Mazowiecka 15, 92-215 Lodz, Poland; 2 Department of Cardiac Surgery, Medical University of Lodz, Lodz, Poland; 3 Department of Pediatrics, Diabetology, Endocrinology and Nephrology, Medical University of Lodz, Lodz, Poland; 4 Department of Clinical Genetics, Medical University of Lodz, Lodz, Poland

**Keywords:** GPR146, Cholesin, Adipose tissue, Diabetes, SGLT2i

## Abstract

**Background:**

Recently deorphanized G protein-coupled receptor 146 (GPR146) was shown to respond to signal from a newly identified hormone—cholesin—and to play a role in hepatic lipid metabolism. However, the importance of its biological activity in human organism remains elusive, mainly due to the lack of studies on human tissues up to this point. This study aimed to identify the cholesin receptor-associated genes and clinical factors linked with their expression in cardiovascular system and associated adipose tissues.

**Methods:**

Right cardiac auricle, aortic wall, saphenous vein, and adipose tissue (periaortic-PAT, epicardial-EAT, thymic-TAT) samples were collected during coronary artery bypass grafting. Clinical records of the study participants were assessed for the presence of diabetes, medications taken and serum cholesterol levels. GPR146 mRNA expression in all gathered tissues was assessed with qPCR, and RNA seqencing was performed in selected tissues of 20 individuals to identify pathways associated with GPR146 expression.

**Results:**

We included 46 participants [37 male, 23 with type 2 diabetes, median age 68.50 (Q1–Q3: 63.00–72.00) years, BMI 28.39 (26.06–31.49) kg/m^2^]. GPR146 expression in adipose tissues significantly correlated with BMI, c-peptide, total cholesterol, and LDL concentrations. Selected metabolic pathways were significantly and positively enriched in GPR146-dependent manner. GPR146-coexpressed genes contained key regulators of lipid metabolism involved in such pathways as fatty acid metabolism, tricarboxilic acid cycle and peroxisomal metabolism. Those genes correlated positively with serum concentrations of LDL, HDL, and total cholesterol. SGLT2i treatment was associated with inversion of GPR146-related signature in EAT, suggesting potential impact on cholesin-GPR146 network.

**Conclusions:**

GPR146 expression is associated with serum lipids and metabolically-relevant transcriptomic changes in EAT similar to SGLT2i-associated ones.

**Graphical abstract:**

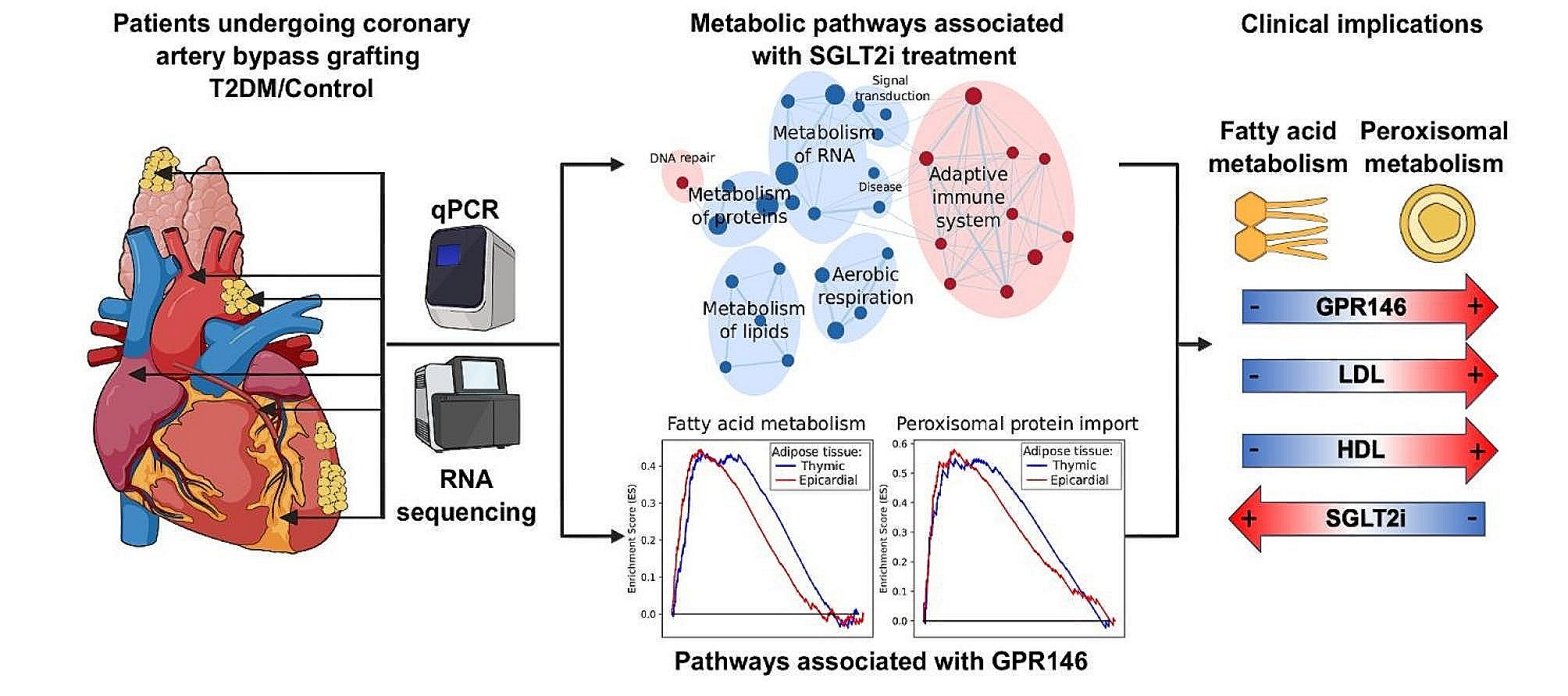

**Supplementary Information:**

The online version contains supplementary material available at 10.1186/s12933-024-02322-y.

## Background

Recently, Hu et al. reported on a new gut-derived hormone, cholesin, whose primary role is to inhibit liver cholesterol synthesis in response to intestinal cholesterol absorption. The team also identified the target receptor mediating cholesin’s effect, GPR146, an orphan receptor allegedly involved in cholesterol liver metabolism pathways [1]. Importantly, cholesin-GPR146 axis holds promise as a therapeutic target for hyperlipidemia and atherosclerosis [2, 3], and the team already hinted at possible synergism with statin treatment [1]. Several studies indicate, that GPR146 impacts cholesterol metabolism in the liver via the ERK1/2 and SREBP2 signalling pathways. Depletion of GPR146 results in decreased levels of total cholesterol and triglycerides, emphasizing its potential role in atherosclerosis development [3]. Moreover, other studies revealed, that GPR146 may be important in vascular remodelling, along with adipose tissue development and metabolism [4, 5]. SGLT2 inhibitors (SGLT2i) are routinely prescribed drugs to T2DM patients with cardiovascular disease, though theirs cardioprotective effects are not fully elucidated [6]. Hypotheses include the beneficial impact of decreased volemic load due to iSGLT2, anti-inflammatory properties or a direct influence of SGLT2i on cardiomyocytes. Though this theory is debatable, due to lack of strong evidence of SGLT2 expression in cardiac tissue [7–9].

In this communication, we bring attention to the GPR146-related pathways in diabetes and their synergies with cardioprotective therapies such as SGLT2i. We hypothesized that pathways downstream from GPR146 are active in cardiovascular-associated adipose tissues and may be affected by treatment of diabetes with SGLT2i.

## Methods

The study was approved by the Bioethics Committee of the Medical University of Lodz (RNN/187/21/KE).

Participants were recruited between 2022–2023 at the Department of Cardiac Surgery of the Medical University of Lodz. Adults with type 2 diabetes (T2DM) and with significant left main coronary artery stenosis or multivessel disease qualified for elective coronary artery bypass grafting (CABG) were recruited to the study. Exclusion criteria included: severe chronic kidney disease (eGFR < 30 ml/1.73 m^2^), chronic liver disease (C/D in Child–Pugh classification) or use of sulphonylureas.

**Fig. 1:**
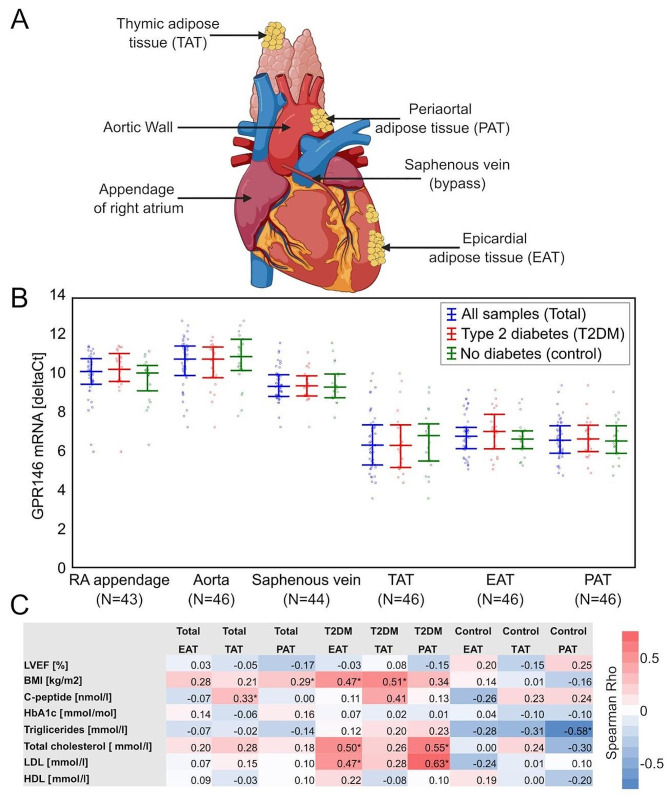
Associations of GPR146 expression data in studied tissues with clinical data. **A** Sources of tissues collected during the surgeries (Adapted from “Coronary artery bypass graft” template by Biorender.com (2024). Retrieved from https://app.biorender.com/biorender-templates); **B** Expression levels of GPR146 measured using qPCR in the whole group (blue) and individuals with (red), or without (green) diabetes. Values are represented as dCt—higher values represent lower relative expression levels. Expression was uniformly similar across all three types of adipose tissue and significantly greater than in the blood vessel and myocardium samples. **C** Heatmap of correlation coefficients of GPR146 expression in Epicardial (EAT), Thymic (TAT) and Periaortal (PAT) adipose tissues. Significant (p < 0.05) correlations were marked with asterisk (*). *LVEF* left ventricle ejection fraction, *BMI* body mass index, *TG* triglycerides, *LDL* low-density lipoprotein, *HDL* high-density lipoprotein, *HbA1c* glycated hemoglobin

Tissue samples were extracted during surgery from right cardiac auricle, aortic wall, saphenous vein, and adipose tissues [thymic (TAT), epicardial (EAT), and periaortic (PAT), Fig. 1A]. Tissue samples were placed in RNAlater (Thermo Fisher Scientific, Waltham, MA, USA) immediately after collection and stored in − 80 °C until analysis.

The following clinical data were collected: age, BMI, T2DM treatment, serum concentration of total cholesterol, low-density lipoprotein (LDL), high-density lipoprotein (HDL), triglycerides, glycated haemoglobin (HbA1c) and fasting c-peptide.

### Real-time quantitative polymerase chain reaction (qPCR)

Tissue samples were mechanically homogenized with magnetic beads in RLT buffer (Qiagen, Germany) with addition of β-Mercaptoethanol. RNA was isolated with RNAeasy minikit (Qiagen, Germany) according to the manufacturer’s protocol. The mRNA expression of *GPR146* and reference gene *GAPDH* were analysed using TaqMan® Assays (#402,869, Thermo Fisher Scientific, Waltham, MA, USA) on LighCycler480 system (Roche, Switzerland).

### RNA-sequencing

Libraries were constructed using QIAseq Stranded mRNA Library Kit (Qiagen, Germany) following the manufacturer’s protocol. The RNAseq procedure was performed with NextSeq 500/550 High Output Kit v2.5 (300 Cycles; Illumina, California, USA) following the protocol provided by the manufacturer.

### Data analysis

Raw data was acquired from Illumina BaseSpace (Illumina, California, USA) using GenerateFASTQ v.2.0.1. FASTQ files were processed using nf-core/rnaseq pipeline v.3.14.0 [10] following standard procedure. After adapter trimming with TrimGalore v.0.6.7 and cutadept v.3.4 and deduplication with Picard v.3.0.0, reads were aligned to the human reference genome (GRCh38.p13) and counts obtained using Salmon v.1.10.1 and STAR v.2.6.1d, with *homo sapiens* NCBI version 108 GTF annotation file. Read counts were normalized to the transcripts per million (TPM). Detailed methods, raw and processed data are available in Gene Expression Omnibus (GSE263644). Due to non-normal distribution of plasma lipid parameters we used nonparametric Mann–Whitney’s and Spearman’s rank correlation test for qPCR data analysis. We considered results with P values < 0.05 for single comparisons as significant, while for Gene Set Enrichment Analysis (GSEA) [11] a Benjamini-Hochberg-adjusted threshold (False Discovery Rate—FDR) of < 0.15 was established. Enrichment plots were drawn using GSEApy and matplotlib, and enrichment maps using Cytoscape [12]. The sample size was restricted by patient availability and material quality, but the number of 46 individuals (balanced 23:23 between patients with and without diabetes) allowed us to identify tissues with correlations between GPR146 levels and lipid concentrations with a magnitude of r >|0.4| with power of 80% at a p level < 0.05. For within-group correlations, statistical power of 80% was maintained for correlations with r >|0.55| at p < 0.05 representing biologically strong and potentially clinically-relevant effects. Choice of tissues for miRNA sequencing was based on the observed pattern of associations.

## Results

We included 46 participants [37 (80.4%) male, 23 (50%) with T2DM, median age 68.50 (Q1–Q3: 63.00–72.00) years, BMI 28.39 (26.06–31.49) kg/m^2^]. Patients with T2DM were treated in line with national guidelines for diabetes management at that time, majority with SGLT2i, metformin, and/or insulin (details in Supplementary Fig. 1). Besides HbA1c, only HDL concentration differed between those with or without T2DM (1.01 [0.91–1.21] vs 1.30 [0.97–1.69], p = 0.0425, Table 1).

**Table 1:** Basic clinical characteristics of patients with and without diabetes included in the study

Variable	Total (N = 46) Me (Q1–Q3)	T2DM (N = 23) Me (Q1–Q3)	Control (N = 23) Me (Q1–Q3)	p-value
Age [years]	68.50 (63.00–72.00)	68.00 (64.00–72.00)	69.00 (60.00–72.00)	0.4588
BMI [kg/m^2^]	28.39 (26.06–31.49)	29.37 (27.85–31.61)	27.18 (24.78–31.49)	0.1422
Triglicerides [mmol/l]	1.38 (1.07–2.12)	1.48 (1.00–2.45)	1.27 (1.17–1.82)	0.4857
Total cholesterol [mmol/l]	4.07 (3.13–4.96)	3.99 (3.07–4.26)	4.61 (3.28–5.19)	0.1679
LDL [mmol/l]	2.44 (1.89–3.37)	2.27 (1.84–2.66)	2.88 (1.95–3.65)	0.1044
HDL [mmol/l]	1.06 (0.94–1.39)	1.01 (0.91–1.21)	1.30 (0.97–1.69)	**0.0425**
C-peptide [nmol/l]	1.24 (0.81–1.78)	1.10 (0.81–1.78)	1.28 (0.80–2.27)	0.4327
HbA1c [mmol/mol]	40.00 (37.00–51.00)	51.00 (45.00–62.00)	38.00 (34.00–40.00)	** < 0.0001**
LVEF [%]	50.00 (45.00–55.00)	48.00 (45.00–54.00)	50.00 (47.00–56.00)	0.4074

p value in bold indicates statistical significance (p<0.05)

*T2DM* type 2 diabetes, *BMI* body mass index, *LDL* low density lipoprotein, *HDL* high-density lipoprotein, *HbA1c* glycated hemoglobin, *LVEF* left ventricle ejection fraction

Expression of GPR146 was confirmed in all six sampled tissues, with the EAT, TAT and PAT showing ~ 16-fold greater levels than the vessels and myocardium (Fig. 1B). No significant differences were noted depending on the presence of T2DM, but among individuals with T2DM significant correlations were observed for EAT GPR146 levels and BMI, LDL and total cholesterol (Fig. 1C).

**Fig. 2:**
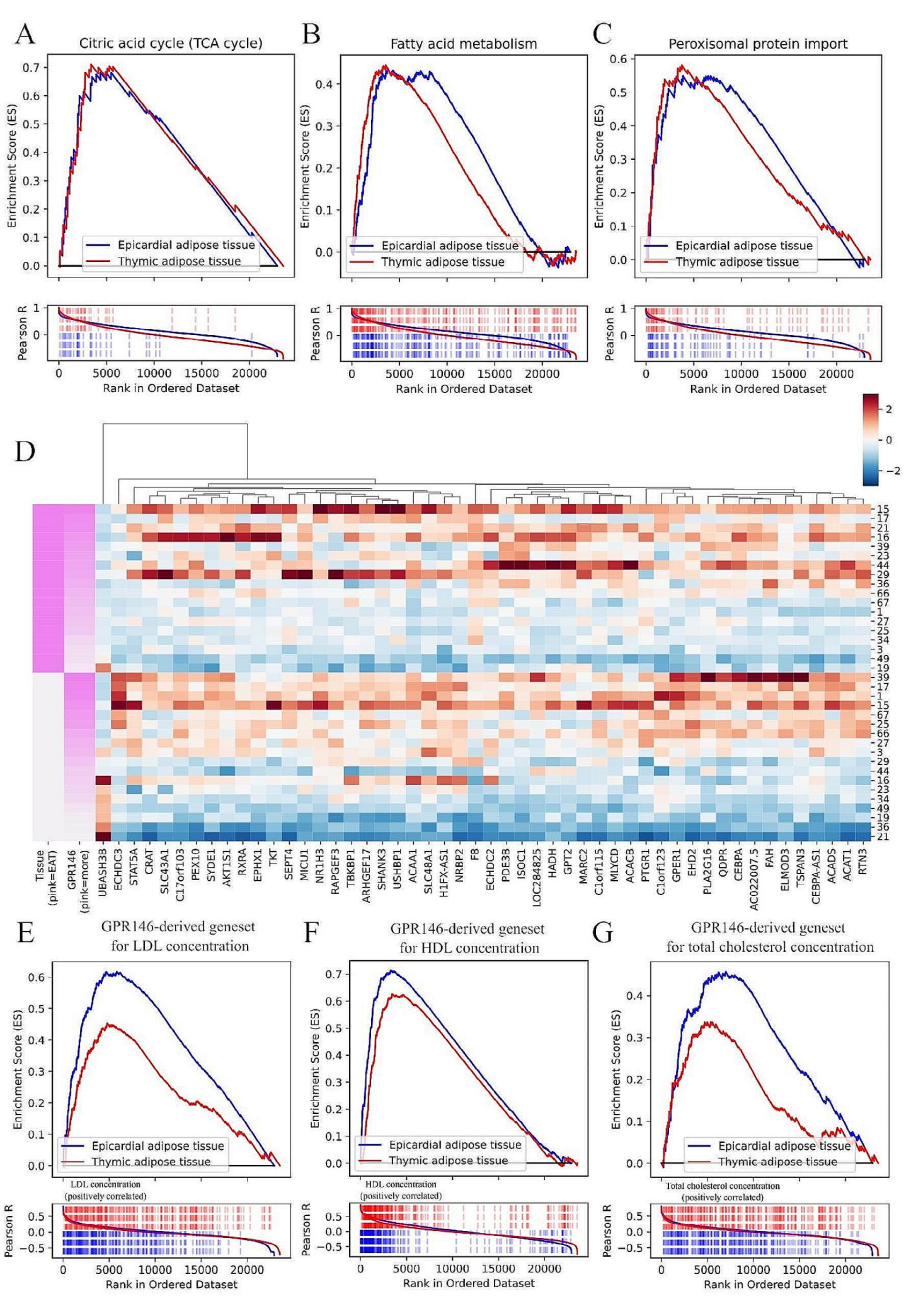
Gene expression landscape in RNA-sequencing data of epicardial (EAT) and thymic (TAT) adipose tissues. **A**–**C** Enrichment plots of selected genesets enrichment in EAT (blue) and TAT (red): citric acid TCA cycle (**A**), fatty acid metabolism (**B**) and peroxisomal protein transport pathways (**C**). **D** Heatmap of gene expression (z-score, normalized within gene) of 50 genes with the strongest correlation in both EAT and TAT with GPR146 levels. Pink tissue class marker represents EAT and white TAT. **E**–**G** Enrichment plots of GPR146-associated geneset which correlated significantly in both EAT and TAT with: LDL (**E**), HDL (**F**) and total cholesterol levels (**G**)

Similarly, in PAT, GPR146 expression level strongly correlated with total cholesterol and LDL exclusively in patients with T2DM. A distinct profile was observed in TAT, which showed a significant correlation between GPR146 expression and fasting c-peptide, but no association with cholesterol levels (details for all tissues in Supplementary Table 1).These contrasting characteristics guided our choice of EAT and TAT, metabolically-distinct tissues, for RNA sequencing to extend our perspective on cholesin–GPR146 network in adipose tissue. We selected a subset of matched 10:10 individuals with and without T2DM for RNA-seq analysis. We provided clinical characteristics of the RNA-seq group and its subset of patients with T2DM in Supplementary Tables 2 and 3. GSEA showed a substantial number of genesets as significantly correlated with GPR146 in both EAT and TAT (Supplementary Table 4). The strongest associations were observed for fatty acid metabolism, tricarboxylic acid cycle, and peroxisomal protein import (Fig. 2A–C) as well as multiple pathways associated with mitochondrial processes (Supplementary Figs. 2 and 3). The transcriptomic profiles and geneset-GPR146 associations in EAT and TAT showed overlap for 36 (15.93%) up- and 34 (6.84%) down-regulated pathways (Supplementary Fig. 4). All this led us to conclude that GPR146 belongs to a metabolically active signaling pathway in cardiovascular system-associated adipose tissues. Finally, we identified genes with the strongest association with GPR146 levels (|R|> 0.60 in both EAT and TAT; Supplementary Table 5) as a putative GPR146-associated geneset (Fig. 2D and Supplementary Fig. 5). Among those genes we identified crucial regulators of lipid metabolism (e.g. CEBPA [13], CD36 [14]) suggesting strong associations of GPR146 signaling with atherogenic processes and lipid metabolism. This was evident on the clinical level, where the GPR146-associated geneset activity correlated positively and significantly with LDL, HDL and total cholesterol levels (Fig. 2E–G).

**Fig. 3:**
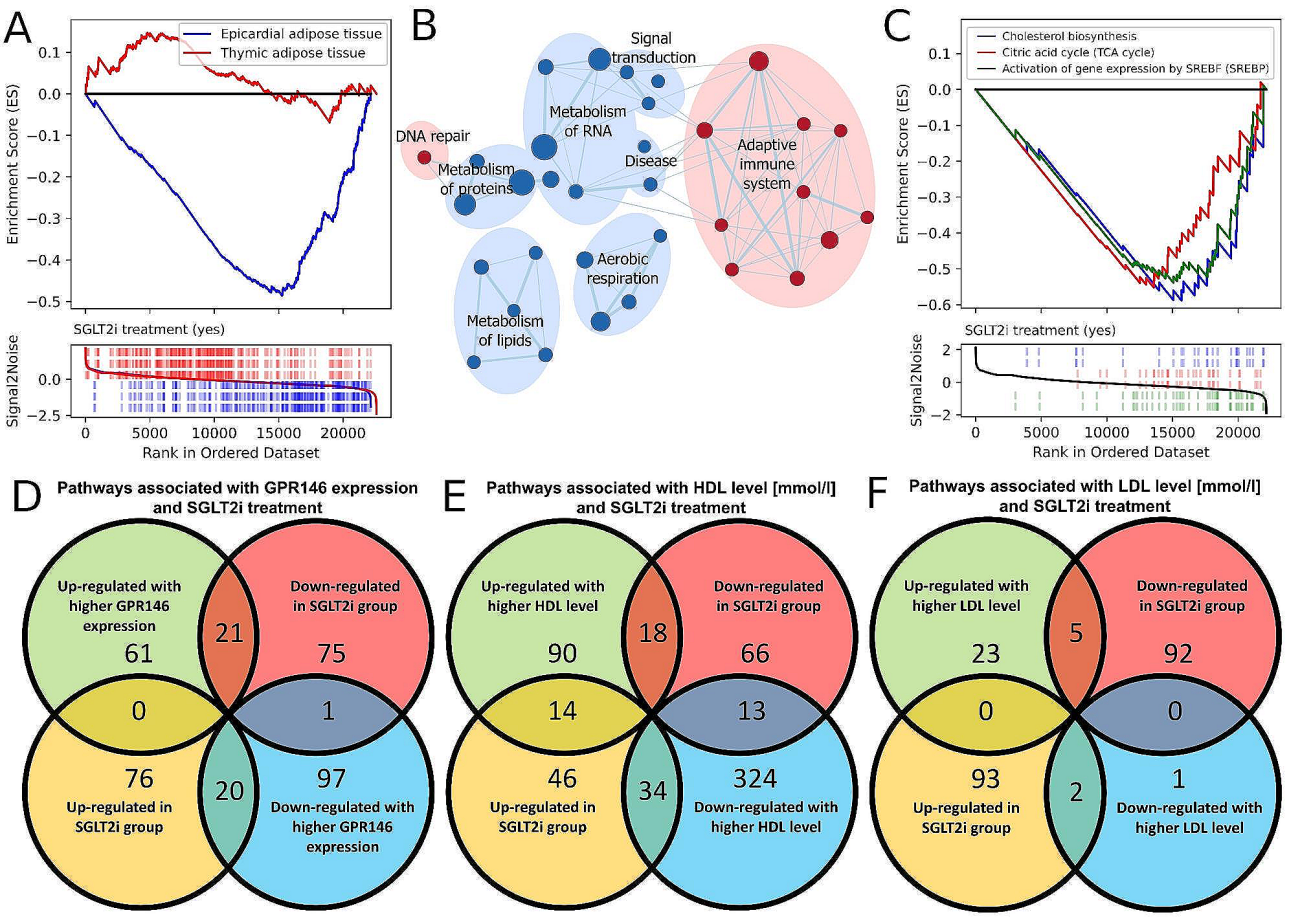
**A** Enrichment plot of the GPR146-associated geneset which showed significant downregulation in SGLT2-treated individuals in EAT but not in TAT. **B** Enrichment map of pathways significantly associated with SGLT2i treatment. **C** Enrichment plot of significant suppression of cholesterol biosynthesis (blue), citric acid cycle (TCA cycle) (red) and activation of gene expression of by SREBF (SREBP) (green) signaling pathways in EAT in patients treated with SGLT2i. **D**–**F** Venn diagrams of pathways significantly associated with SGLT2i treatment and GPR146 expression (p < 0.0001), HDL (p < 0.0001) or LDL (p = 0.0022)

Despite a limited sample size, we were able to show that SGLT2i treatment was associated with significant downregulation of GPR146-associated geneset, specifically in EAT (Fig. 3A). To investigate this further we evaluated how SGLT2i exposure affected the transcriptomic profiles in EAT and identified significant down-regulation of pathways associated with cholesterol biosynthesis, TCA cycle and activation of gene expression by SREBF (Fig. 3B, C). Strikingly, geneset expression patterns associated with SGLT2i were inverse to ones significantly correlated with GPR146 expression (p < 0.0001), HDL and (p < 0.0001) LDL (p = 0.0022) (Fig. 3D–F; Supplementary Table 4). On a gene-wise level exposure to SGLT2i treatment was associated with lower expression levels of major regulatory elements of lipid metabolism: PPARG, CEBPA, SREBF2 and cholesin itself (Supplementary Table 6) in EAT with an average decrease by 20% of expression and no discernible effect in TAT although the differences did not reach statistical significance due to the limited number of patients in this subgroup analysis.

## Discussion

Our data expands on the recent findings of Hu et al. and strongly suggests that the role of cholesin-GPR146 signaling in the body may be far greater than initially assumed. The original report suggested that the cholesin receptor is present and functional in hepatocytes [1]. However, prior sources that investigated GPR146 before the identification of its ligand, demonstrated that its mRNA expression is also abundant across other tissues, including ones affected by diabetes complications [4, 15]. Our study showed the presence of GPR146 expression in aorta, saphenous vein, appendage of right atrium as well as abundant presence in PAT, EAT and TAT. Moreover, we demonstrated strong correlations between GPR146 expression and serum lipids in perivascular adipose tissues. These associations were absent in vessels themselves,which suggests that a regulatory network is active at adipocyte level specifically. Consequently, it is likely that the EAT and PAT both exhibit at least some form of regulation through cholesin and GPR146. In contrast to other adipose tissues, the association between GPR146 expression and the level of lipid parameters was not visible in TAT. This may be due to a different metabolic profile of this tissue type. EAT and PAT are known to be highly metabolically active tissues with well-described paracrine activity [16], whereas little is known about the physiological role of TAT. TAT development is generally considered to be an effect of aging process although there is some evidence that it serves as a source of humoral and angiogenic factors used for angiogenic processes in myocardium [17]. Noneoftheless, TAT’s physiology seems very different to EAT and PAT, hence it may not be a primary target tissue for cholesterol-driven signaling response affecting GPR146 in particular.

The results of this study reveal that GPR146-associated signalling is not restricted to the liver, but it is also important in cardiovascular system-associated adipose tissues. Pathways downstream of GPR146 and a cascade of events on the scale of human organism triggered by this complex remain to be explored. At this point it is unknown if and how GPR146 activation and signalling in adipocytes is linked to intestine-hepatocytes axis. It is possible that just like in hepatocytes, GPR146-cholesin signalling in adipocytes causes a decrease in cholesterol biosynthesis, although adipose tissue is not the dominant source of cholesterol. It is also likely that GPR146 signalling regulates some other metabolic mechanisms like adipogenesis and uptake of free cholesterol to eliminate its excess in order orchestrate cholesterol homeostasis. The connection between the role of cholesin-GPR146 in hepatocytes and adipocytes is thus an intriguing subject for further studies.

An even more interesting observation is the association between the expression of GPR146-associated network of genes with serum lipids (which confirms the qPCR data) and its dependence on treatment with SGLT2i. Some of the effects were not consistent between the qPCR and RNA-seq analysis. A notable example was the absence of correlation between GPR146 and total cholesterol or LDL in qPCR, whereas GSEA analysis showed the association of the GPR146-pathway with HDL and LDL. Most likely this is due to the greater power of the GSEA analysis in identifying a cumulative effect stemming from a large number of weak, gene-level associations. Undoubtedly, this necessitates an in-depth mechanistical investigation and external validation in other settings.

The impact of SGLT2i on lipid levels is widely known with reports showing increased rates of lipolysis and elevated plasma levels of large LDL fraction and HDL cholesterol [18–20]. Animal model studies aimed to explain these effects through inhibition of peroxisomal factors like DGAT2, PPARG1 and PPARG2, but no clear mechanism linking the SGLT2i to these pathways was thus far identified [21–23]. Furthermore, reduction of epicardial fat volumes after SGLT2i treatment was described consistently across multiple studies [3, 24, 25], but the exact mechanism remained elusive. Here, we show that peroxisomal pathways are associated with GPR146 expression and suppressed by SGLT2i treatment. Due to the limited number of patients treated with iSGLT2 in Poland (current national guidelines list metformin as first line therapy [26]) the observation is one of very limited power and in need of external validation. While our results do not show conclusively how SGLT2i could affect EAT metabolism and volume, we show that genes strongly correlated at transcriptional level with GPR146 were affected by SGLT2i treatment. This finding warrants further investigation and highlights the importance of this newly-defined receptor-ligand pair and their signaling pattern. If this result is confirmed, the impact of SGLT2i on plasma lipids may be attributable to this regulatory network explaining for the first time some of the observable effects of this drug class. While in-depth mechanistic investigations of the cholesin-GPR146 network could not be reliably performed in tissues from patients undergoing cardiovascular surgeries that we investigated, this is the first report to demonstrate that the activity of this ligand-receptor pair is likely not restricted to the intestine-liver axis and shows strong associations with a number of important clinical metabolic traits and aspects of diabetes treatment. Most importantly, our data show that GPR146 associated signalling is likely important for the metabolic activity of cardiovascular system-associated adipose tissues and thus potentially linked to atherogenesis and cardiac outcomes. If these effects can be exploited through treatment with cholesin-like ligands, the clinical implications may be far-reaching.

## Conclusions

Expression levels of GPR146 in tissues associated with diabetic complications show strong associations with lipid profiles and metabolic traits. Metabolic pathways downstream of GPR146 are active in cardiovascular system-associated adipose tissue and are likely suppressed by SGLT2i treatment.

### Supplementary Information

Supplementary material 1: Figure 1 Summary of medications used for treatment of diabetes.

Supplementary material 2: Figure 2 Enrichment map of pathways significantly associated with GPR146 expression levels in EAT.

Supplementary material 3: Figure 3 Enrichment map of pathways significantly associated with GPR146 expression levels in TAT.

Supplementary material 4: Figure 4 Venn diagram of pathways significantly up- and down-regulated in EAT and TAT.

Supplementary material 5: Figure 5 Heatmap of all genes that correlated with GPR146 expression with R >|0.60| in EAT and TAT.

Supplementary material 6: Figure 6 Enrichment map of pathways significantly associated with SGLT2i in EAT.

Supplementary material 7: Figure 7 Consent to participate declaration form used during the recruitment process.

Supplementary material 8: Supplementary Table 1.

Supplementary material 9: Supplementary Table 2.

Supplementary material 10: Supplementary Table 3.

Supplementary material 11: Supplementary Table 4.

Supplementary material 12: Supplementary Table 5.

Supplementary material 13: Supplementary Table 6.

## Data Availability

Raw sequencing data was uploaded to the SRA and GEO repositories under the Accession Number GSE263644. Raw qPCR data is available from the Authors upon reasonable request.

## Abbreviations

GPR146: G protein-couple receptor 146
PAT: Peroaortic adipose tissue
EAT: Epicardial adipose tissue
TAT: Thymic adipose tissue
qPCR: Real-time quantitative polymerase chain reaction
RNA-seq: RNA sequencing
BMI: Body mass index
LDL: Low-density lipoprotein
HDL: High-density lipoprotein
SGLT2i: Sodium-glucose contransporter-2 inhibitors
T2DM: Type 2 diabetes mellitus
CABG: Coronary artery bypass grafting
eGFR: Estimated glomerular filtration rate
HbA1c: Glycated haemoglobin
GSEA: Gene set enrichment analysis
FDR: False discovery rate
TPM: Transcripts per million
CEBPA: CCAAT/enhancer-binding protein-alpha
CD36: Cluster of differentiation 36
IGF1: Insulin-like growth factor 1
DGAT2: Diacylglycerol O-acyltransferase 2
PPARG1: Peroxisome proliferator activated receptor gamma 1
PPARG2: Peroxisome proliferator activated receptor gamma 2

## References

[1] Hu X, Chen F, Jia L, Long A, Peng Y, Li X (2024). A gut-derived hormone regulates cholesterol metabolism. Cell.

[2] Han F, Liu X, Chen C, Liu Y, Du M, Zhou Y (2020). Hypercholesterolemia risk-associated GPR146 is an orphan G-protein coupled receptor that regulates blood cholesterol levels in humans and mice. Cell Res.

[3] Yu H, Rimbert A, Palmer AE, Toyohara T, Xia Y, Xia F (2019). GPR146 deficiency protects against hypercholesterolemia and atherosclerosis. Cell.

[4] Huang J, Xie Y, Chen B, Xia Y, Jiang Y, Sun Z (2023). GPR146 regulates pulmonary vascular remodeling by promoting pulmonary artery smooth muscle cell proliferation through 5-lipoxygenase. Eur J Pharmacol.

[5] Kaczmarek I, Wower I, Ettig K, Kuhn CK, Kraft R, Landgraf K (2023). Identifying G protein-coupled receptors involved in adipose tissue function using the innovative RNA-seq database FATTLAS. iScience.

[6] Seidu S, Alabraba V, Davies S, Newland-Jones P, Fernando K, Bain SC (2024). SGLT2 inhibitors—the new standard of care for cardiovascular, renal and metabolic protection in type 2 diabetes: a narrative review. Diabetes Therapy.

[7] Marfella R, Scisciola L, D’Onofrio N, Maiello C, Trotta MC, Sardu C (2022). Sodium-glucose cotransporter-2 (SGLT2) expression in diabetic and non-diabetic failing human cardiomyocytes. Pharmacol Res.

[8] D’Onofrio N, Sardu C, Trotta MC, Scisciola L, Turriziani F, Ferraraccio F (2021). Sodium-glucose co-transporter2 expression and inflammatory activity in diabetic atherosclerotic plaques: effects of sodium-glucose co-transporter2 inhibitor treatment. Mol Metab.

[9] Clementi E, Corbi G, Boccardi V, Scisciola L (2022). Anti-inflammatory role of SGLT2 inhibitors as part of their anti-atherosclerotic activity: data from basic science and clinical trials. Front Cardiovasc Med.

[10] Di Tommaso P, Chatzou M, Floden EW, Barja PP, Palumbo E, Notredame C (2017). Nextflow enables reproducible computational workflows. Nat Biotechnol.

[11] Subramanian A, Tamayo P, Mootha VK, Mukherjee S, Ebert BL, Gillette MA (2005). Gene set enrichment analysis: a knowledge-based approach for interpreting genome-wide expression profiles. Proc Natl Acad Sci USA.

[12] Shannon P, Markiel A, Ozier O, Baliga NS, Wang JT, Ramage D (2003). Cytoscape: a software environment for integrated models of biomolecular interaction networks. Genome Res.

[13] Lefterova MI, Zhang Y, Steger DJ, Schupp M, Schug J, Cristancho A (2008). PPARγ and C/EBP factors orchestrate adipocyte biology via adjacent binding on a genome-wide scale. Genes Dev.

[14] Li Y, Huang X, Yang G, Xu K, Yin Y, Brecchia G (2022). CD36 favours fat sensing and transport to govern lipid metabolism. Prog Lipid Res.

[15] Uhlén M, Fagerberg L, Hallström BM, Lindskog C, Oksvold P, Mardinoglu A, et al. Tissue-based map of the human proteome. Science (1979). 2015;347(6220). 10.1126/science.1260419 25613900

[16] Iacobellis G (2022). Epicardial adipose tissue in contemporary cardiology. Nat Rev Cardiol.

[17] Tinahones F, Salas J, Mayas MD, Ruiz-Villalba A, Macias-Gonzalez M, Garrido-Sanchez L (2009). VEGF gene expression in adult human thymus fat: a correlative study with hypoxic induced factor and cyclooxigenase-2. PLoS ONE.

[18] Szekeres Z, Toth K, Szabados E (2021). The effects of sglt2 inhibitors on lipid metabolism. Metabolites.

[19] Nagao M, Sasaki J, Tanimura-Inagaki K, Sakuma I, Sugihara H, Oikawa S (2024). Ipragliflozin and sitagliptin differentially affect lipid and apolipoprotein profiles in type 2 diabetes: the SUCRE study. Cardiovasc Diabetol.

[20] Basu D, Huggins LA, Scerbo D, Obunike J, Mullick AE, Rothenberg PL (2018). Mechanism of increased LDL and decreased triglycerides with SGLT2 inhibition HHS public access. Arterioscler Thromb Vasc Biol.

[21] Ji W, Zhao M, Wang M, Yan W, Liu Y, Ren S (2017). Effects of canagliflozin on weight loss in high-fat diet-induced obese mice. PLoS ONE.

[22] Packer M (2022). Critical reanalysis of the mechanisms underlying the cardiorenal benefits of SGLT2 inhibitors and reaffirmation of the nutrient deprivation signaling/autophagy hypothesis. Circulation.

[23] Hoong CWS, Chua MWJ (2021). SGLT2 inhibitors as calorie restriction mimetics: insights on longevity pathways and age-related diseases. Endocrinology (United States).

[24] Masson W, Lavalle-Cobo A, Nogueira JP (2021). Effect of sglt2-inhibitors on epicardial adipose tissue: a meta-analysis. Cells.

[25] Cinti F, Leccisotti L, Sorice GP, Capece U, D’Amario D, Lorusso M (2023). Dapagliflozin treatment is associated with a reduction of epicardial adipose tissue thickness and epicardial glucose uptake in human type 2 diabetes. Cardiovasc Diabetol.

[26] Araszkiewicz A, Bandurska-Stankiewicz E, Borys S (2022). Guidelines on the management of patients with diabetes: a position of diabetes poland. Curr Top Diabetes.

