# Supplementary figures and images for: Cholesin receptor signalling is active in cardiovascular system-associated adipose tissue and correlates with SGLT2i treatment in patients with diabetes
Source: Cardiovasc Diabetol. 2024 Jun 20;23:211. doi: 10.1186/s12933-024-02322-y (PMC11191148; doi:10.1186/s12933-024-02322-y)

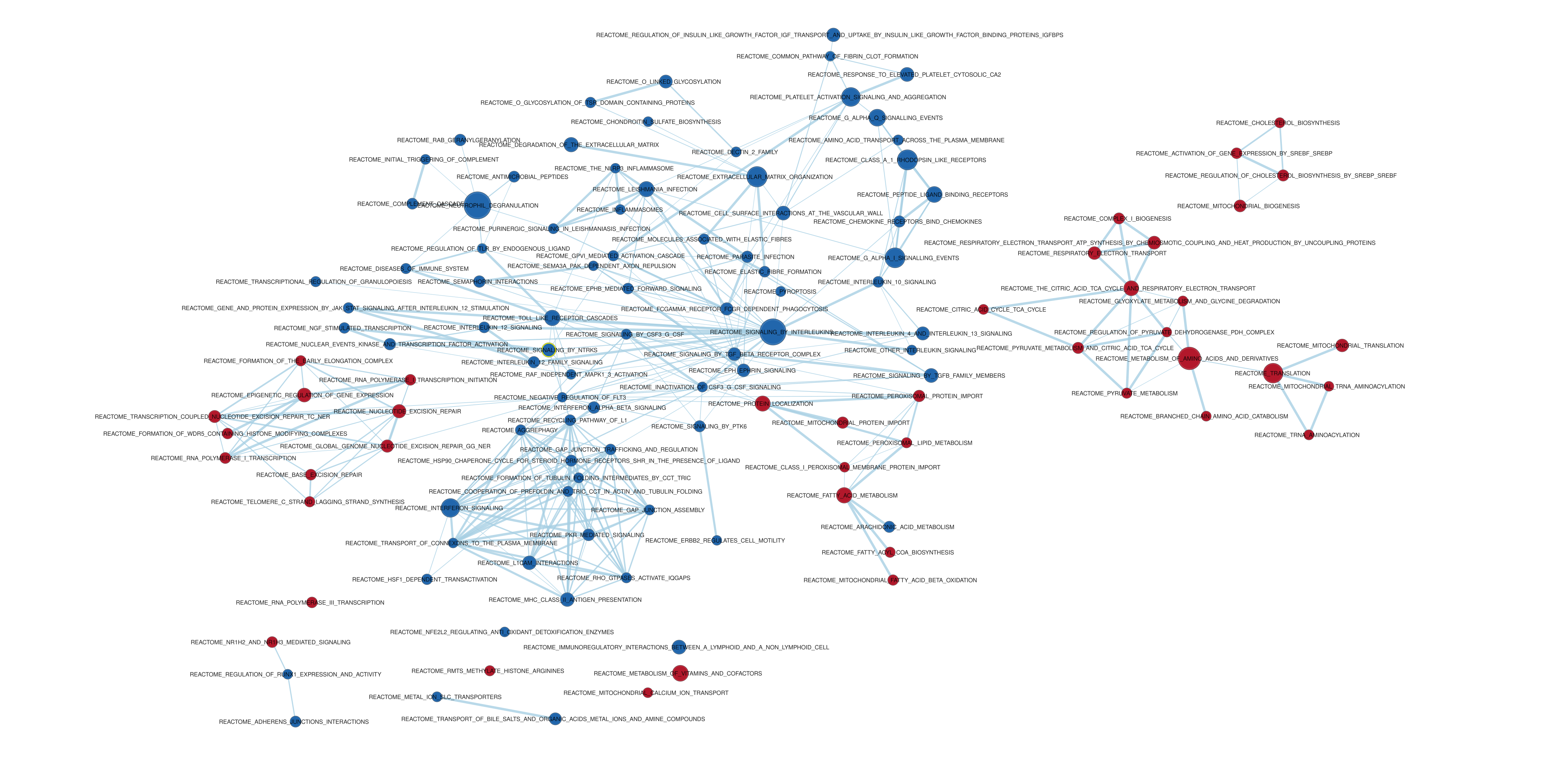

Supplement: Supplementary file 2 — Supplementary material 2: Figure 2 Enrichment map of pathways significantly associated with GPR146 expression levels in EAT. [file 12933_2024_2322_MOESM2_ESM.tiff]

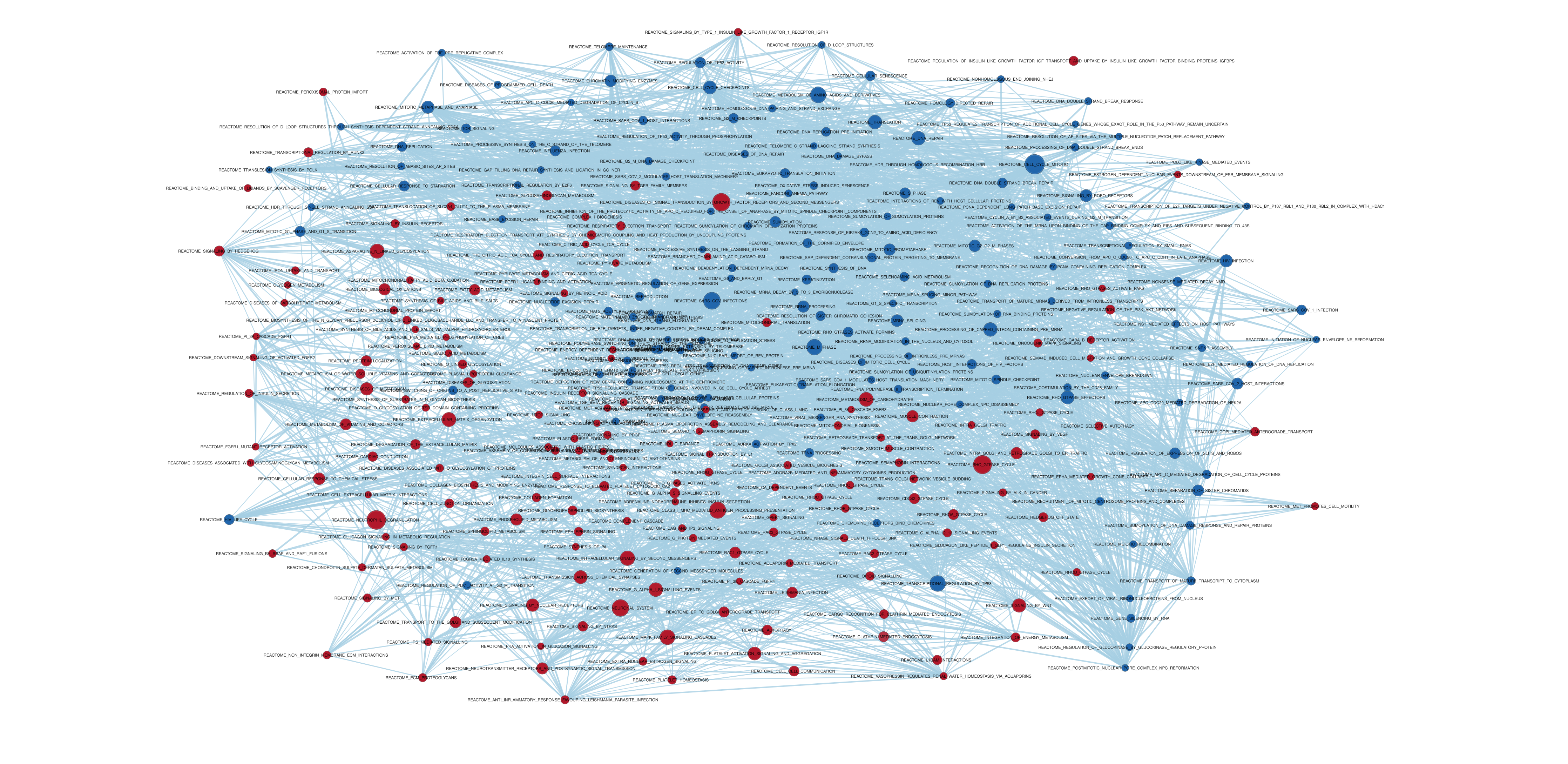

Supplement: Supplementary file 3 — Supplementary material 3: Figure 3 Enrichment map of pathways significantly associated with GPR146 expression levels in TAT. [file 12933_2024_2322_MOESM3_ESM.tiff]

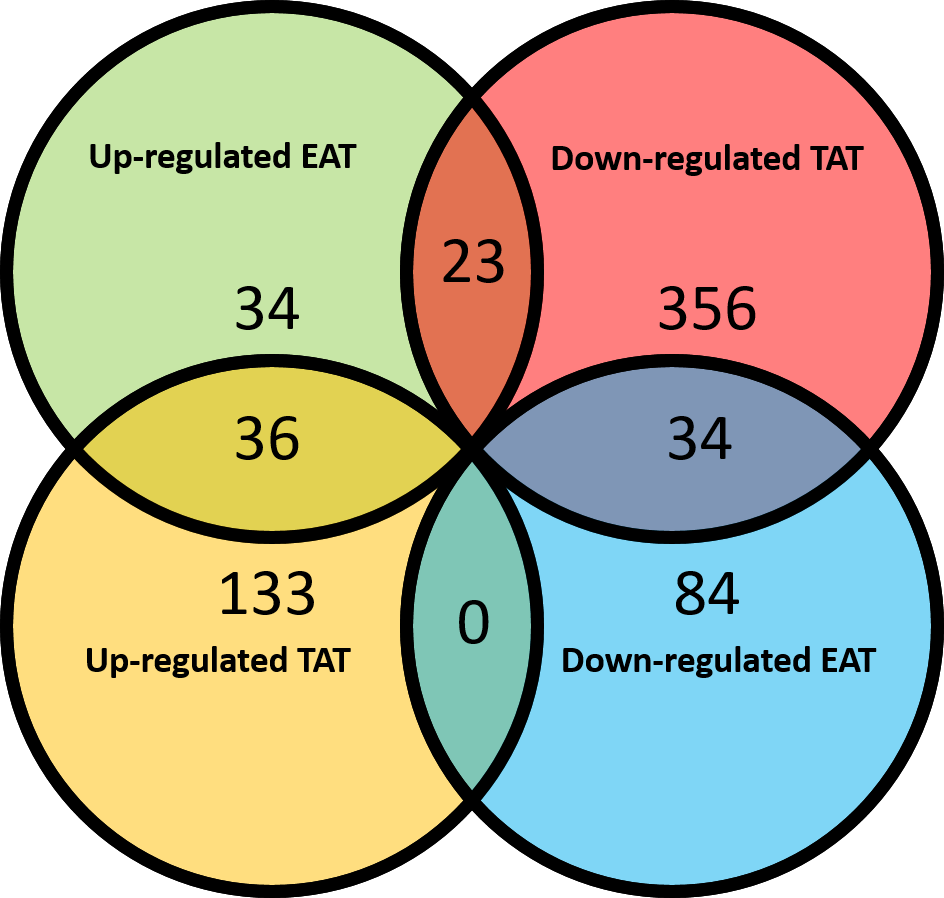

Supplement: Supplementary file 4 — Supplementary material 4: Figure 4 Venn diagram of pathways significantly up- and down-regulated in EAT and TAT. [file 12933_2024_2322_MOESM4_ESM.tiff]

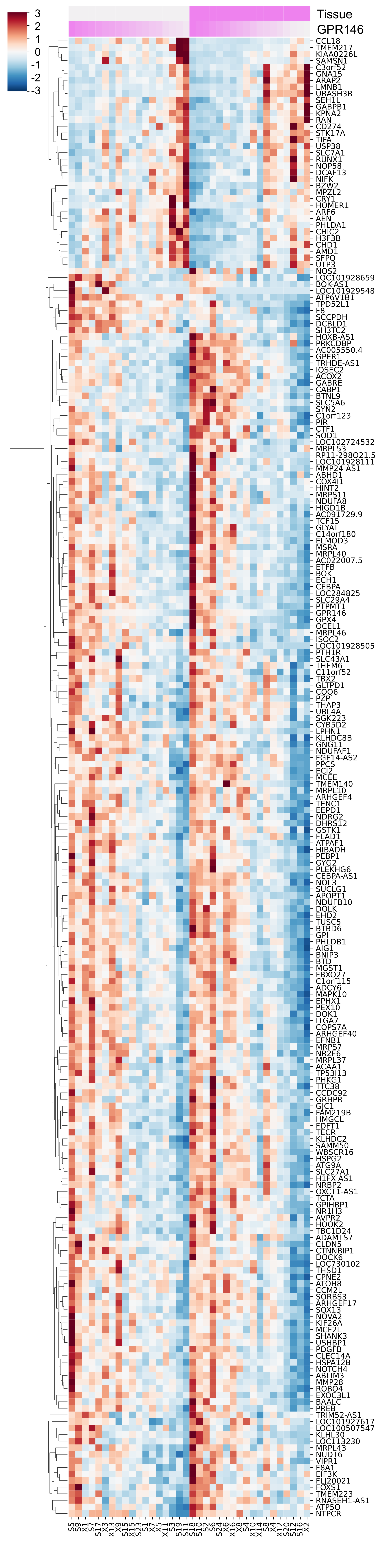

Supplement: Supplementary file 5 — Supplementary material 5: Figure 5 Heatmap of all genes that correlated with GPR146 expression with R >|0.60| in EAT and TAT. [file 12933_2024_2322_MOESM5_ESM.tiff]

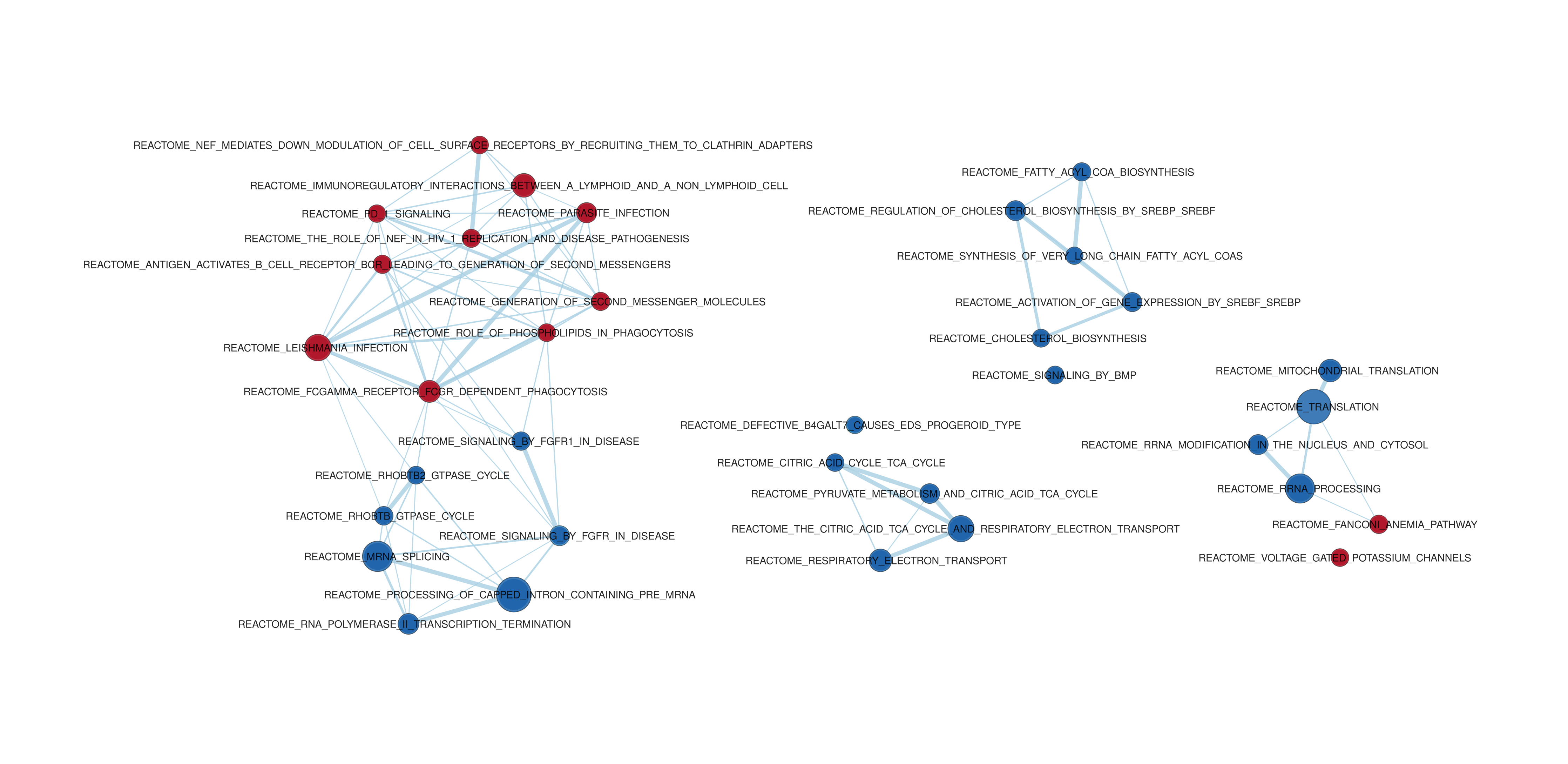

Supplement: Supplementary file 6 — Supplementary material 6: Figure 6 Enrichment map of pathways significantly associated with SGLT2i in EAT. [file 12933_2024_2322_MOESM6_ESM.tiff]
