# Supplementary material for: Cholesin receptor signalling is active in cardiovascular system-associated adipose tissue and correlates with SGLT2i treatment in patients with diabetes
Source: Cardiovasc Diabetol. 2024 Jun 20;23:211. doi: 10.1186/s12933-024-02322-y (PMC11191148; doi:10.1186/s12933-024-02322-y)
Supplement: Supplementary file 7 — Supplementary material 7: Figure 7 Consent to participate declaration form used during the recruitment process. [file 12933_2024_2322_MOESM7_ESM.pdf]

Patient consent form

For participation in the study entitled: **Investigating the mechanism of action of C-peptide on cholesterol metabolism in patients with diabetes**

I (name and surname).., (date of birth).......................... declare that I have been made aware of the content of the study information. The investigator has provided me with comprehensive answers to my questions. I have had the opportunity to ask questions and obtain additional information before making a decision. I am sufficiently informed about the aims and methods of the study. In case of any questions or doubts that arise during the study I have the possibility to contact the principal investigator or co-researchers through the office of the Department of Biostatistics and Translational Medicine at the Medical University of Lodz (15 Mazowiecka Street, 92-215 Lodz).

I knowingly and voluntarily consent to the use of my biological material from the right atrial appendage, saphenous vein, aortic wall, thymic, periaortic and epicardial adipose tissue, which will be collected during the coronary artery bypass grafting surgery. I also consent to the use of venous blood taken from me in the diagnostic and treatment process for the study. I consent to my medical records being used for the purposes of the above study.

I know that I can withdraw my consent at any time without affecting the medical care that will be required.

Łódź, date ______________________

Patients's signature: _________________________

Phone number ______________________________

Signature of the attending physician ______________________________
