# Supplementary material for: Cholesin receptor signalling is active in cardiovascular system-associated adipose tissue and correlates with SGLT2i treatment in patients with diabetes
Source: Cardiovasc Diabetol. 2024 Jun 20;23:211. doi: 10.1186/s12933-024-02322-y (PMC11191148; doi:10.1186/s12933-024-02322-y)
Supplement: Supplementary file 8 — Supplementary material 8: Supplementary Table 1. [file 12933_2024_2322_MOESM8_ESM.docx]

**Supplementary Table 1** – Correlation coefficients between *GPR146* expression and clinical variables

| **Variable** | | **BMI**  **[kg/m^2^]** | **C-peptide [nmol/l]** | **HbA1c [%]** | **Total cholesterol[mmol/l]** | **TG [mmol/l]** | **LDL [mmol/l]** | **HDL [mmol/l]** | **LVEF [%]** |
| --- | --- | --- | --- | --- | --- | --- | --- | --- | --- |
| **RA appendage** | Total (N=49) | **R=0.32; p=0.034** | R=0.07; p=0.632 | R=0.04; p=0.8092 | R=0.21; p=0.1759 | R=0.08; p=0.614 | R=0.07; p=0.6517 | R=0.09; p=0.5667 | R=-0.04; p=0.7766 |
|  | T2DM (N=23) | **R=0.50; p=0.0173** | R=0.03; p=0.8791 | R=0.06; p=0.7867 | R=0.33; p=0.1277 | R=0.04; p=0.8731 | R=0.27; p=0.2295 | R=0.36; p=0.0967 | R=0.09; p=0.6934 |
|  | Control (N=26) | R=0.10; p=0.6786 | R=0.12; p=0.6037 | **R=-0.47; p=0.0310** | R=0.20; p=0.3830 | R=0.12; p=0.5958 | R=-0.04; p=0.8778 | R=0.07; p=0.7689 | R=-0.11; p=0.6250 |
| **Aorta** | Total (N=52) | **R=0.32; p=0.0293** | R=0.04; p=0.8002 | R=-0.10; p=0.5168 | R=0.17; p=0.2644 | R=0.05; p=0.7265 | R=0.06; p=0.6731 | R=0.13; p=0.3963 | R=0.06; p=0.6797 |
|  | T2DM (N=24) | R=0.37; p=0.0844 | R=-0.08; p=0.6999 | R=0.22; p=0.3096 | R=0.14; p=0.5113 | R=0.13; p=0.5590 | R=0.21; p=0.3397 | R=0.07; p=0.7674 | R=0.15; p=0.5060 |
|  | Control (N=28) | R=0.37; p=0.0809 | R=0.11; p=0.6086 | R=-0.20; p=0.3497 | R=0.09; p=0.6799 | R=-0.01; p=0.9536 | R=-0.11; p=0.6246 | R=0.14; p=0.5200 | R=-0.06; p=0.8007 |
| **Saphenous vein** | Total (N=50) | R=0.08; p=0.6112 | R=0.11; p=0.4700 | R=0.10; p=0.5096 | R=0.15; p=0.3356 | R=-0.03; p=0.8630 | R=0.05; p=0.7239 | R=0.24; p=0.1220 | R=0.09; p=0.5591 |
|  | T2DM (N=23) | R=-0.11; p=0.6400 | R=0.30; p=0.1786 | R=0.27; p=0.2329 | R=0.16; p=0.4743 | R=0.20; p=0.3834 | R=0.14; p=0.5426 | R=-0.11; p=0.6203 | R=0.03; p=0.9003 |
|  | Control (N=27) | R=0.17; p=0.4570 | R=-0.13; p=0.5641 | R=0.02; p=0.9156 | R=0.17; p=0.4385 | R=-0.23; p=0.3127 | R=0.01; p=0.9483 | **R=0.54; p=0.0094** | R=0.18; p=0.4262 |
| **TAT** | Total (N=52) | R=0.21; p=0.1649 | **R=0.33; p=0.0253** | R=-0.06; p=0.6706 | R=0.28; p=0.0621 | R=-0.02; p=0.9164 | R=0.15; p=0.3301 | R=-0.03; p=0.8346 | R=-0.05; p=0.7200 |
|  | T2DM (N=24) | **R=0.51; p=0.0125** | R=0.41; p=0.0539 | R=0.02; p=0.9124 | R=0.26; p=0.2329 | R=0.20; p=0.3598 | R=0.28; p=0.1881 | R=-0.08; p=0.7199 | R=0.08; p=0.7303 |
|  | Control (N=28) | R=0.01; p=0.9643 | R=0.23; p=0.2850 | R=-0.10; p=0.6371 | R=0.24; p=0.2737 | R=-0.31; p=0.1555 | R=0.01; p=0.9607 | R=0.00; p=0.9821 | R=-0.15; p=0.4969 |
| **EAT** | Total (N=52) | R=0.28; p=0.0622 | R=-0.07; p=0.6308 | R=0.14; p=0.3587 | R=0.20; p=0.1910 | R=-0.07; p=0.6652 | R=0.07; p=0.6645 | R=0.09; p=0.5496 | R=0.03; p=0.8484 |
|  | T2DM (N=24) | **R=0.47; p=0.0232** | R=0.11; p=0.6183 | R=0.07; p=0.7633 | **R=0.50; p=0.0158** | R=0.12; p=0.5713 | **R=0.47; p=0.0238** | R=0.22; p=0.3034 | R=-0.03; p=0.9068 |
|  | Control (N=28) | R=0.14; p=0.5261 | R=-0.26; p=0.2383 | R=0.04; p=0.8724 | R=0.00; p=0.9946 | R=-0.28; p=0.1894 | R=-0.24; p=0.2686 | R=0.19; p=0.3896 | R=0.20; p=0.3568 |
| **PAT** | Total (N=52) | **R=0.29; p=0.0474** | R=0.00; p=0.9822 | R=0.16; p=0.2991 | R=0.18; p=0.2296 | R=-0.14; p=0.3442 | R=0.10; p=0.4919 | R=0.10; p=0.5217 | R=-0.17; p=0.2568 |
|  | T2DM (N=24) | R=0.34; p=0.1081 | R=0.13; p=0.5470 | R=0.01; p=0.9624 | **R=0.55; p=0.0063** | R=0.23; p=0.2862 | **R=0.63; p=0.0011** | R=0.10; p=0.6455 | R=-0.15; p=0.5001 |
|  | Control (N=28) | R=0.25; p=0.2558 | R=-0.16; p=0.4557 | R=0.24; p=0.2633 | R=-0.10; p=0.6569 | **R=-0.58; p=0.0039** | R=-0.30; p=0.1593 | R=0.10; p=0.6472 | R=-0.20; p=0.3532 |

RA – right auricle, TAT – thymic adipose tissue, EAT – epicardial adipose tissue, PAT – periaortic adipose tissue. T2DM – type 2 diabetes, BMI – body mass index, LDL – low density lipoprotein, HDL – high-density lipoprotein, HbA1c – glycated hemoglobin, LVEF – left ventricle ejection fraction
