# Supplementary material for: Cholesin receptor signalling is active in cardiovascular system-associated adipose tissue and correlates with SGLT2i treatment in patients with diabetes
Source: Cardiovasc Diabetol. 2024 Jun 20;23:211. doi: 10.1186/s12933-024-02322-y (PMC11191148; doi:10.1186/s12933-024-02322-y)
Supplement: Supplementary file 9 — Supplementary material 9: Supplementary Table 2. [file 12933_2024_2322_MOESM9_ESM.docx]

**Supplementary Table 2 –** Clinical characteristics of patients in whom RNA sequencing was performed in EAT and TAT.

| **Variable** | **Total (N=20)** | **T2DM (N=10)** | **Control (N=10)** | **p-value** |
| --- | --- | --- | --- | --- |
|  | **N (%)** | **N (%)** | **N (%)** |  |
| **Sex (Male)** | 17 (75%) | 8 (80%) | 7 (70%) | 1.0000 |
|  | **Me (Q1-Q3)** | **Me (Q1-Q3)** | **Me (Q1-Q3)** |  |
| **Age [years]** | 68.00  (63.00 - 69.50) | 68.00  (64.00 - 70.00) | 68.50  (58.00 - 69.00) | 0.5027 |
| **BMI [kg/m^2^]** | 28.14  (25.90 - 30.97) | 28.39  (26.47 - 30.45) | 27.84  (24.74 - 31.10) | 0.0713 |
| **Triglicerides [mmol/l]** | 1.31  (1.01 - 2.05) | 1.49  (1.20 - 2.45) | 1.22  (1.00 - 1.35) | 0.2969 |
| **Total cholesterol [mmol/l]** | 4.21  (3.38 - 5.50) | 4.01  (3.30 - 4.24) | 5.06  (4.09 - 5.80) | 0.7009 |
| **LDL [mmol/l]** | 2.66  (2.06 - 3.40) | 2.45  (1.97 - 2.66) | 3.39  (2.45 - 4.06) | 0.0025 |
| **HDL [mmol/l]** | 1.19  (0.99 - 1.44) | 1.10  (0.89 - 1.22) | 1.35  (1.05 - 1.54) | 0.0018 |
| **C-peptide [nmol/l]** | 1.04  (0.76 - 1.91) | 1.04  (0.73 - 2.03) | 1.10  (0.80 - 1.61) | 0.1022 |
| **HbA1c [mmol/mol]** | 40.00  (38.00 - 49.90) | 49.90  (43.20 - 51.00) | 38.00  (36.60 - 40.00) | 0.3431 |
| **LVEF [%]** | 50.00  (44.50 - 52.50) | 48.00  (44.00 - 52.00) | 50.00  (48.00 - 54.00) | 0.3478 |
| **T2DM treatment (N=10)** | |  | | |
| **Metformin** | | 4 (40%) | NA | NA |
| **Metformin+Insulin** | | 1 (10%) | NA | NA |
| **Metformin and SGLT2i** | | 5 (50%) | NA | NA |

EAT – epicardial adipose tissue, TAT – thymic adipose tissue, BMI – body mass index, LDL – low density lipoprotein, HDL – high-density lipoprotein, HbA1c – glycated hemoglobin, LVEF – left ventricle ejection fraction
