# Supplementary material for: Cholesin receptor signalling is active in cardiovascular system-associated adipose tissue and correlates with SGLT2i treatment in patients with diabetes
Source: Cardiovasc Diabetol. 2024 Jun 20;23:211. doi: 10.1186/s12933-024-02322-y (PMC11191148; doi:10.1186/s12933-024-02322-y)
Supplement: Supplementary file 10 — Supplementary material 10: Supplementary Table 3. [file 12933_2024_2322_MOESM10_ESM.docx]

Supplementary Table 3 – Clinical characteristics of T2DM patients whose samples were evaluated using RNA-sequencing depending on treatment with SGLT2i+metformin or metformin alone*.

| **Variable** | **Total**  **(N=10)** | **Metformin + SGLT2i (N=5)** | **Metformin**  **(N=5)** | **p-value** |
| --- | --- | --- | --- | --- |
|  | **N (%)** | **N (%)** | **N (%)** |  |
| **Sex (Male)** | 8 (80%) | 5 (100%) | 3 (60%) | 0.4444 |
|  | **Me (Q1-Q3)** | **Me (Q1-Q3)** | **Me (Q1-Q3)** |  |
| **Age [years]** | 68.00  (64.00 - 70.00) | 68.00  (63.00 - 70.00) | 68.00  (67.00 - 69.00) | 0.8413 |
| **BMI [kg/m^2^]** | 28.39  (26.47 - 30.45) | 28.37  (27.02 - 30.45) | 28.41  (25.32 - 28.73) | 0.6905 |
| **Triglicerides [mmol/l]** | 1.49  (1.20 - 2.45) | 2.45  (1.20 - 2.81) | 1.48  (1.34 - 1.49) | 0.5476 |
| **Total cholesterol [mmol/l]** | 4.01  (3.30 - 4.24) | 3.99 (3.01 -  4.03) | 4.17  (3.46 - 4.24) | 0.4206 |
| **LDL [mmol/l]** | 2.45  (1.97 - 2.66) | 2.46 (1.84 -  2.65) | 2.43  (2.15 - 2.66) | 0.6905 |
| **HDL [mmol/l]** | 1.10  (0.89 - 1.22) | 0.95 (0.89 -  1.21) | 1.16  (1.04 - 1.30) | 0.4206 |
| **C-peptide [nmol/l]** | 1.04  (0.73 - 2.03) | 1.11  (0.96 - 2.25) | 0.84  (0.73 - 1.78) | 0.4206 |
| **HbA1c [mmol/mol]** | 49.90  (43.20 - 51.00) | 50.80 (49.00 - 51.00) | 46.00  (43.20 - 51.00) | 0.6905 |
| **LVEF [%]** | 48.00  (44.00 - 52.00) | 48.00 (48.00 - 52.00) | 45.00  (44.00 - 53.00) | 1.0000 |

BMI – body mass index, LDL – low density lipoprotein, HDL – high-density lipoprotein, HbA1c – glycated hemoglobin, LVEF – left ventricle ejection fraction

* One individual received a low dose of long-acting basal insulin alongside metformin treatment.
