# Supplementary material for: Cholesin receptor signalling is active in cardiovascular system-associated adipose tissue and correlates with SGLT2i treatment in patients with diabetes
Source: Cardiovasc Diabetol. 2024 Jun 20;23:211. doi: 10.1186/s12933-024-02322-y (PMC11191148; doi:10.1186/s12933-024-02322-y)
Supplement: Supplementary file 13 — Supplementary material 13: Supplementary Table 6. [file 12933_2024_2322_MOESM13_ESM.docx]

Supplementary Table 6 - Comparison of expression levels of key regulatory genes for lipid metabolism and cholesin (*c7orf50*) in SGLT2i-treated and non-treated patients in EAT and TAT.

|  | **EAT (N=20)** | | | **TAT (N=20)** | | |
| --- | --- | --- | --- | --- | --- | --- |
| **Gene** | **SGLT2i (yes) (N=5)** | **SGLT2i (no) (N=15)** | **P value** | **SGLT2i (yes) (N=5)** | **SGLT2i (no) (N=15)** | **P value** |
| *PPARG* | 6.89 (0.46) | 7.34 (0.41) | 0.0524 | 6.83 (0.71) | 6.44 (1.24) | 0.5108 |
| *CEBPA* | 6.32 (0.47) | 6.72 (0.62) | 0.2124 | 6.46 (0.65) | 6.05 (1.14) | 0.4572 |
| *SREBF2* | 3.49 (0.46) | 3.72 (0.41) | 0.2963 | 3.54 (0.40) | 3.78 (0.43) | 0.2872 |
| *C7orf50* | 5.40 (0.43) | 5.71 (0.32) | 0.1049 | 5.50 (0.27) | 5.58 (0.31) | 0.6150 |

EAT – epicardial adipose tissue, TAT – thymic adipose tissue, SD- standard deviation

Values presented as means +/- standard deviations of log2(TPM)
